# Syntheses and crystal structures of two di­naph­tho[2,1-*d*:1′,2′-*f*][1,3]dithiepine atropisomers

**DOI:** 10.1107/S2056989023000476

**Published:** 2023-01-24

**Authors:** Neil Beare, Gavin F. Painter, C. John McAdam

**Affiliations:** a BDG Synthesis, PO Box 38627, Wellington Mail Centre 5045, Wellington, New Zealand; b Ferrier Research Institute, Victoria University of Wellington, PO Box 33436, Lower Hutt 5046, New Zealand; c Department of Chemistry, University of Otago, PO Box 56, Dunedin 9054, New Zealand; University of Aberdeen, United Kingdom

**Keywords:** crystal structure, atropisomer, bi­naphthalene di­thiol, asymmetric synthesis, hydrogen bonds, C—H⋯π contacts

## Abstract

The closely related title compounds, 1-(di­naphtho­[2,1-*d*:1′,2′-*f*][1,3]dithiepin-4-yl)-2,2-di­methyl­propan-1-ol, C_26_H_24_OS_2_, **1** and 2-(di­naphtho­[2,1-*d*:1′,2′-*f*][1,3]dithiepin-4-yl)-3,3-di­methyl­butan-2-ol, C_27_H_26_OS_2_, **2**, both comprise an atrop­isomeric binaphthyl di­thio­acetal unit substituted at the methyl­ene carbon atom with a chiral neopentyl alcohol grouping. The overall stereochemistry of the racemate in each case is defined as *aS,R* and *aR,S*. In **1**, the hydroxyl group generates inversion dimers *via* pairwise inter­molecular O—H⋯S hydrogen bonds whereas in **2**, the O—H⋯S link is intra­molecular. Weak C—H⋯π inter­actions link the mol­ecules into extended arrays in both structures.

## Abstract 2

The mol­ecular and crystal structures of 1-(di­naphtho­[2,1-*d*:1′,2′-*f*][1,3]dithiepin-4-yl)-2,2-di­methyl­propan-1-ol and 2-(di­naphtho­[2,1-*d*:1′,2′-*f*][1,3]dithiepin-4-yl)-3,3-di­methyl­butan-2-ol, from asymmetric syntheses are reported.

## 1. Chemical context

Stereoselective synthetic methodology continues to be a major research focus underpinning many areas of chemical and biological sciences. One design strategy is the utilization of atropisomerism. This exploits the stereoisomerism that results from restricted rotation about single bonds, a particular feature of biaryl compounds (Cen *et al.*, 2022 ▸; Wencel-Delord *et al.*, 2015 ▸; Cheng *et al.*, 2021 ▸). Di­naphtho­[2,1-*d*:1′,2′-*f*][1,3]dithiepine (**3**) provides access to an organosulfur-stabilized carbanion. This undergoes nucleophilic addition to prochiral electrophiles producing separable diastereomeric products with varying degrees of diastereoselectivity (Delogu *et al.*, 1991 ▸). Delogu and co-workers, however, report significantly improved diastereoselectivity from nucleophilic attack upon substrates in which the chiral auxiliary (di­naphtho­thiepine) is pre-attached to a (benzaldehyde) stereogenic centre. This work reports the synthesis of the prochiral ketone **5** from a pivaldehyde sourced diastereoisomer mix, and its reduction and methyl­ation reactions that occur with high diastereomeric excess. Single-crystal X-ray structures of 1-(di­naphtho­[2,1-*d*:1′,2′-*f*][1,3]dithiepin-4-yl)-2,2-di­methyl­propan-1-ol, **1**, and 2-(di­naphtho­[2,1-*d*:1′,2′-*f*][1,3]dithiepin-4-yl)-3,3-di­methyl­but­an-2-ol, **2**, confirm the relative stereochemistry of the major isomers.

## 2. Structural commentary

**Figure 1:**
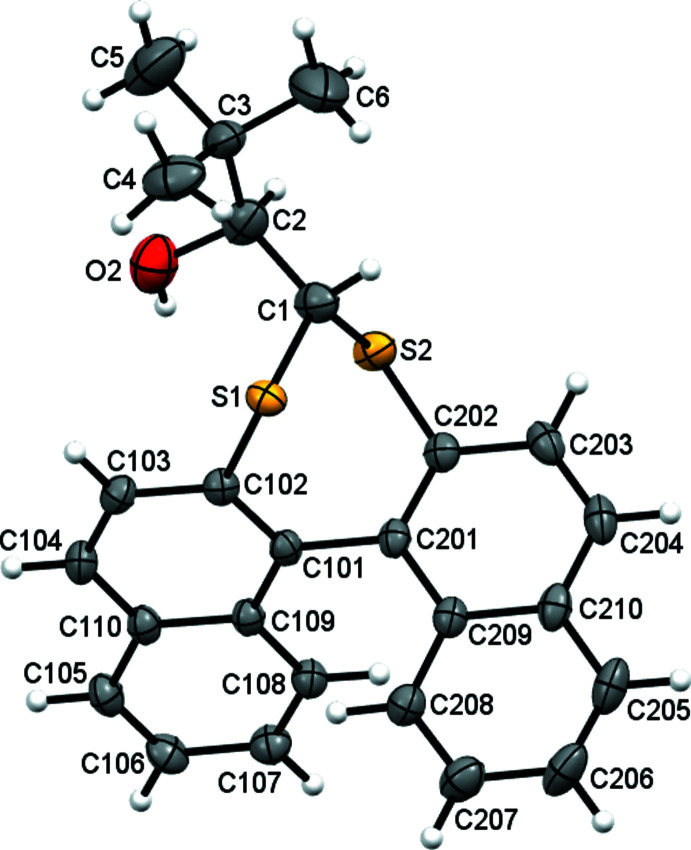
The mol­ecular structure of **1** with displacement ellipsoids drawn at the 50% probability level.

The structural core of compounds **1** and **2** is a 1,1′-linked bi­naphthalene system. This is functionalized at the 2,2′ positions with a disulfaneyl­methane unit, generating a seven-membered ring with pseudo-*C_2_* symmetry locking the bi­naphthalene into *R* and *S* atropisomers. The individual naphthalene ring systems in **1** are predictably flat, with r.m.s. deviations from the ten-atom mean plane of 0.019 and 0.022 Å for C101–C110 and C201–C210 respectively. The C102—C101—C201—C202 torsion angle is −62.5 (3)° and the dihedral angle between naphthalene ring mean planes is 65.91 (4)°. Capping the stereogenic auxiliary is a chiral (at atom C2) neopentyl alcohol group (Fig. 1 ▸), giving *aS,R* and *aR,S* pairs. 
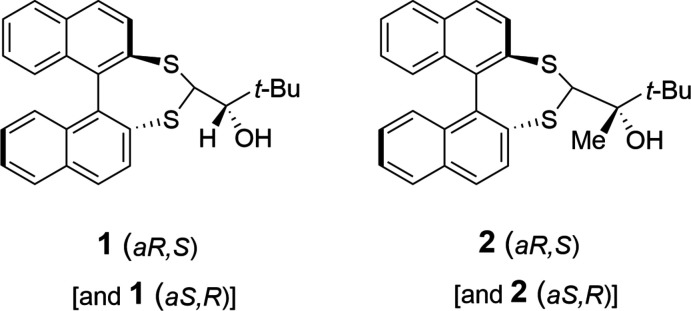


**Figure 2:**
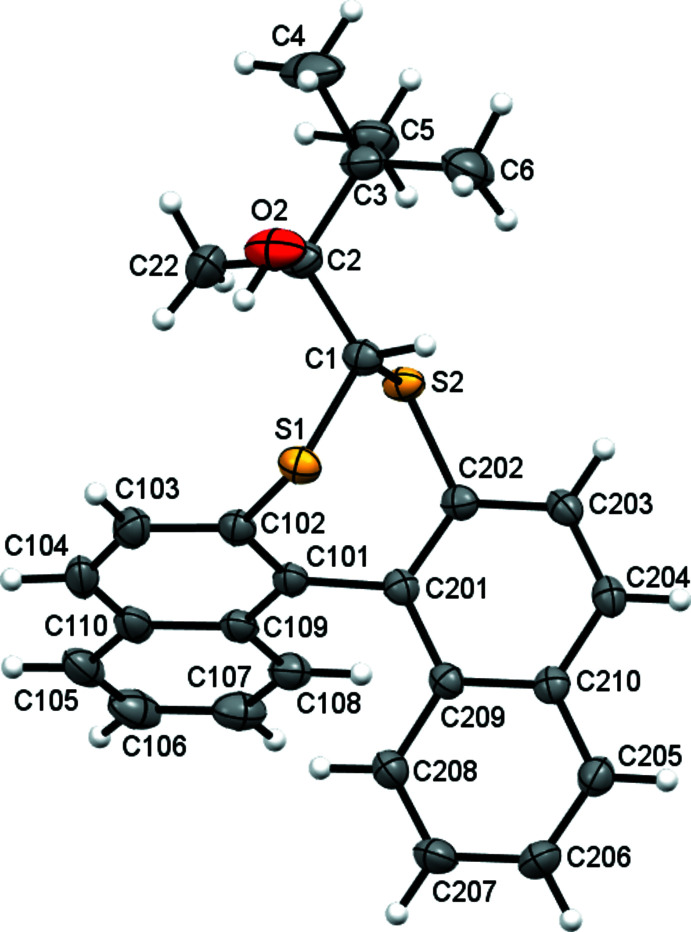
The mol­ecular structure of **2** with displacement ellipsoids drawn at the 50% probability level.

**Figure 3:**
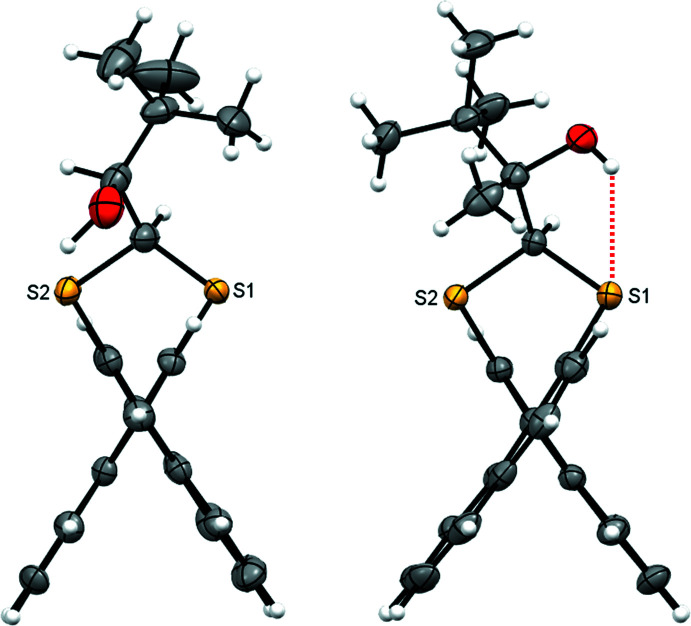
The mol­ecular structures of **1** (left) and **2** (right) aligned with the C101—C201 bond on the *z*-axis. The intra­molecular C—H⋯S bond of **2** is shown as a red dotted line.

The synthesis of compound **2** (Fig. 2 ▸) places a methyl group on the chiral C2 atom in place of the hydrogen atom of **1**. This juxtaposition generates a racemate pair with similar conformation and metrics to **1** (Fig. 3 ▸): r.m.s. deviations from the naphthalene mean planes are 0.05 and 0.04 Å and the C102—C101—C201—C202 torsion angle is −63.95 (19)°, however the dihedral angle between naphthalene rings is larger at 72.35 (3)°. The alcohol group is positioned such to form an intra­molecular hydrogen bond to one of the bridge sulfur atoms (O2—H2*O*⋯S1 = 2.52 Å).

## 3. Supra­molecular features

**Figure 4:**
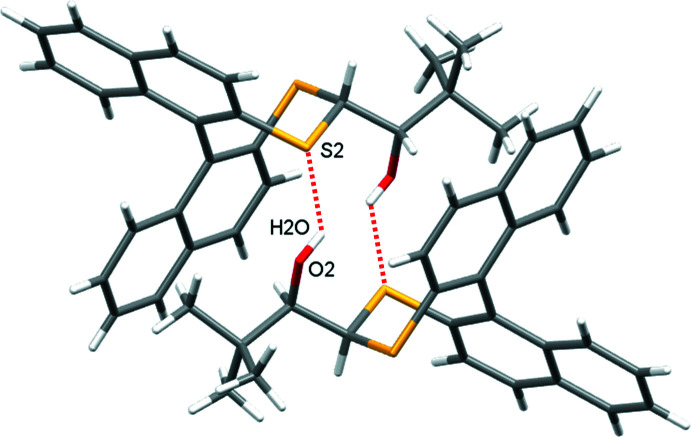
Inversion dimers of **1** formed by pairwise O—H⋯S hydrogen bonds (red dotted lines).

**Figure 5:**
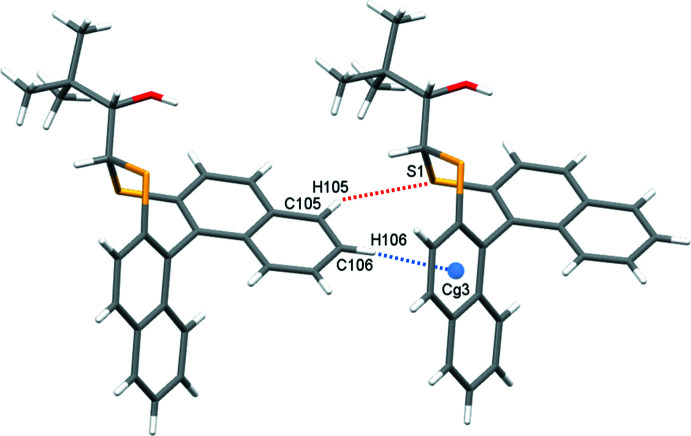
Chains of **1** in the *a*-axis direction formed by C—H⋯π inter­actions (blue dotted lines) and supported with C—H⋯S close contacts (red dotted lines); *Cg*3 is the C201–C204/C210/C209 ring centroid.

**Figure 6:**
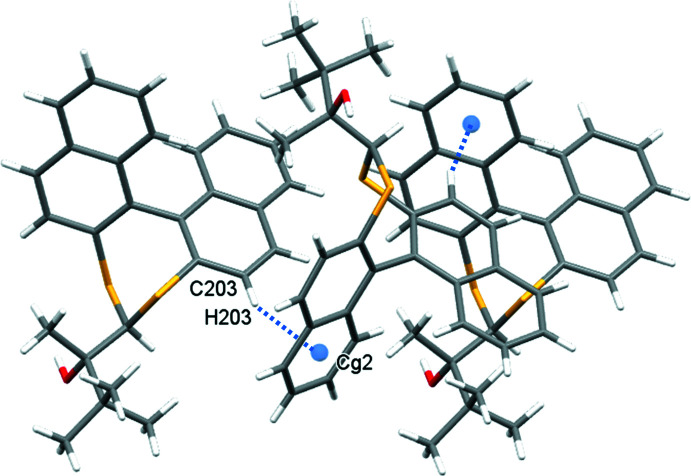
Twofold screw of **2** in the *b*-axis direction formed by C—H⋯π inter­actions (blue dotted lines); *Cg*2 is the C105–C110 ring centroid.

**Figure 7:**
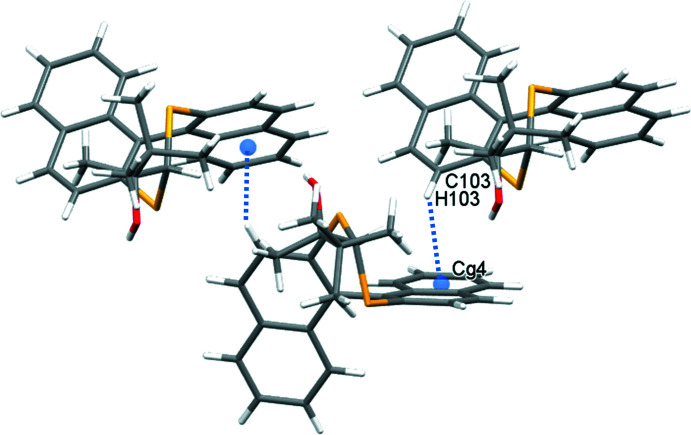
Glide reflection of **2** in the *bc* plane formed by C—H⋯π inter­actions (blue dotted lines); *Cg*4 is the C205–C210 ring centroid.

In the crystal of **1**, inversion dimers form through pairwise classical O2—H2⋯S2 hydrogen bonds (Table 1 ▸), which generate 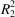 (10) ring motifs (Fig. 4 ▸). C—H⋯π inter­actions between adjacent naphthalene rings link mol­ecules in the *a*-axis direction (C106—H106⋯*Cg*3 = 2.87 Å, where *Cg*3 is the C201–C204/C210/C209 ring centroid). This is supported by a short contact C105—H105⋯S1 of 2.90 Å (Fig. 5 ▸). For **2**, in which the alcohol hydrogen atom is engaged in an intra­molecular hydrogen bond with sulfur (Table 2 ▸), the most important inter­molecular inter­actions are a pair of C—H⋯π inter­actions that propagate in the *b*-axis direction: C203—H203⋯*Cg*2 (2.60 Å) forms a screw diad (Fig. 6 ▸), and C103—H103⋯*Cg*4 (2.93 Å)(*Cg*2 and *Cg*4 are the centroids of the C105–C110 and C205–C210 rings, respectively) forms zigzag chains *via* a glide reflection in the *bc* plane (Fig. 7 ▸).

## 4. Database survey

A search of the Cambridge Structural Database (version 5.41, November 2019 with updates to March 2020; Groom *et al.*, 2016 ▸) suggests the di­naphtho­dithiepine structure is unprecedented, although a di­naphtho­dithiepine *S*-oxide has been reported (refcode JITTEL; Delogu *et al.*, 1991 ▸). The analogous di­naphtho­dioxepine fragment, however, is more common, with more than ten examples reported including the close relative of **1**, 4-(1-meth­oxy-1-phenyl­eth­yl)di­naphtho­[2,1-*d*:1′,2′-*f*][1,3]dioxepine (KUBYEL; Maglioli *et al.*, 1992 ▸), and the simple, but chirally resolved (*R)*-di­naphtho­dioxepine CAJCEY (Zhang *et al.*, 2015 ▸).

## 5. Synthesis and crystallization

**Figure 8:**
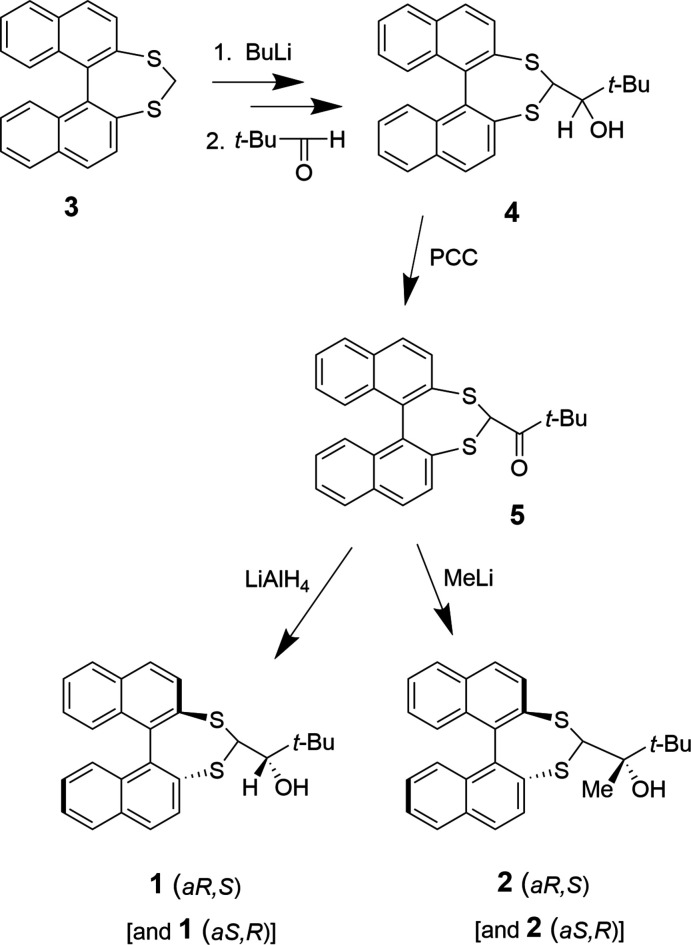
Preparation of **1** and **2**.

Compounds **1** and **2** were synthesized in three steps (Fig. 8 ▸) from dithiepin **3** prepared from a Lewis-acid-catalysed condensation of bi­naphtho­thiol with di­meth­oxy­methane (Delogu *et al.*, 1991 ▸). Diastereoisomer mix (**4**): di­thio­acetal **3** in THF was cooled to 173 K under Ar. *n*-BuLi (1.6 *M* in hexa­nes, 1.2 equiv.) was added dropwise and the suspension stirred for 5 min. Pivaldehyde (1.2 equiv.) was similarly added and the reaction stirred for a further 10 min. The mixture was quenched with saturated NH_4_Cl and extracted with Et_2_O. The ethereal extract was washed, dried (MgSO_4_) and concentrated *in vacuo* then chromatographed (SiO_2_/CH_2_Cl_2_), giving a white solid, a 5:2 mixture of the two possible diastereoisomers of **4** (81%, 44% d.e.). Further chromatography with a CH_2_Cl_2_/hexane solvent allowed separation into major (**1**) and minor (**4_m_**) diastereoisomers. [**4_m_**, m.p. 424 K, ^1^H NMR (200 MHz, CDCl_3_) δ (ppm): 1.02 (9H, *s*, *t*-Bu), 1.90 (1H, *d*, *J* = 6.0 Hz, OH), 4.01 (1H, *dd*, *J* = 6.0, 3.0 Hz, CHOH), 5.18 (1H, *d*, *J* = 3.0 Hz, S—CH—S), 7.07–7.28 (4H, *m*, Ar), 7.42–7.53 (2H, *m*, Ar), 7.80–8.00 (6H, *m*, Ar).]

4-Pivaloyldi­naphtho­[2,1-*d*:1′,2′-*f*][1,3]dithiepine (**5**): to a stirred solution of alcohol **4** in CH_2_Cl_2_ was added CaCO_3_ and powdered 4 Å mol­ecular sieves. PCC (3.3 equiv.) was added and the reaction mix stirred (30 min, Ar, RT). The solvent was concentrated *in vacuo* and filtered through SiO_2_ to give the ketone as a white solid, m.p. 445–446 K (88% yield). ^1^H NMR (200 MHz, CDCl_3_) δ (ppm): 1.30 (9H, *s*, *t*-Bu), 5.73 (1H, *s*, S—CH—S), 7.15 (1H, *d*, *J* = 8.4 Hz, Ar), 7.18–7.31 (3H, *m*, Ar), 7.46–7.56 (2H, *m*, Ar), 7.64 (1H, *d*, *J* = 8.4 Hz, Ar), 7.83 (1H, *d*, *J* = 8.4 Hz, Ar), 7.95–8.03 (4H, *m*, Ar). ^13^C NMR (50 MHz) δ (ppm): 27.2 (Me), 44.3 (CMe_3_), 64.6 (S–CH—S), 126.3, 126.6, 126.8 (Ar CH), 126.9 (Ar C), 127.5, 127.6 (Ar CH), 127.7 (Ar C), 128.3, 128.4, 129.1, 129.2 (Ar CH), 130.4 (Ar C), 132.2 (Ar CH), 131.5 (Ar C), 133.1 (Ar CH), 134.1, 134.5, 142.8, 143.0 (Ar C), 206.2 (C=O).

1-(Di­naphtho­[2,1-*d*:1′,2′-*f*][1,3]dithiepin-4-yl)-2,2-di­methyl­propan-1-ol (**1**): a solution of ketone **5** in THF was cooled to 272 K under Ar. LiAlH_4_ (2 equiv.) was added in one portion and the suspension stirred for 30 min. The reaction mixture was quenched by addition of H_2_O and extracted with Et_2_O. The ethereal extract was washed, dried (MgSO_4_) and concentrated *in vacuo*, then chromatographed (SiO_2_/CH_2_Cl_2_) to give **1** as a white solid, m.p. 426 K (91%, >95% d.e.). Crystals for X-ray diffraction were obtained from slow evaporation of an EtOH/H_2_O solvent mix. ^1^H NMR (300 MHz, CDCl_3_) δ (ppm): 1.01 (9H, *s*, *t*-Bu), 2.70 (1H, *d*, *J* = 6.3 Hz, OH), 3.29 (1H, *dd*, *J* = 6.3, 3.3 Hz, CHOH), 5.16 (1H, *d*, *J* = 3.3 Hz, S—CH—S), 7.10–7.15 (2H, *m*, Ar), 7.18–7.26 (2H, *m*, Ar), 7.44–7.51 (2H, *m*, Ar), 7.80–7.87 (2H, *m*, Ar), 7.90–7.98 (4H, *m*, Ar). ^13^C NMR (75 MHz) δ (ppm): 26.7 (Me), 36.1 (CMe_3_), 70.1 (COH), 80.2 (S—CH—S), 126.5, 126.7, 127.6, 127.7, 128.3 (Ar CH), 128.8 (Ar C), 129.0, 129.2 (Ar CH), 131.5 (Ar C), 132.2 (Ar CH), 132.3 (Ar C), 133.0 (Ar CH), 133.9, 134.0, 141.7, 142.6 (Ar C).

2-(Di­naphtho­[2,1-*d*:1′,2′-*f*][1,3]dithiepin-4-yl)-3,3-di­methyl­butan-2-ol (**2**): a solution of ketone **5** in THF was cooled to 193 K under Ar. MeLi (1.0 *M* in Et_2_O, 5 equiv.) was added and the solution stirred 30 min. The reaction mixture was quenched by addition of EtOD then H_2_O and extracted with Et_2_O. The ethereal extract was washed, dried (MgSO_4_) and concentrated *in vacuo*, then chromatographed on SiO_2_ (1:1 CH_2_Cl_2_/hexa­ne) to give **2** as a white solid, m.p. 448–449 K (81%, >95% d.e.). Crystals for X-ray diffraction were obtained from slow evaporation of an EtOH/H_2_O mix. ^1^H NMR (200 MHz, CDCl_3_) δ (ppm): 1.10 (9H, *s*, *t*-Bu), 1.16 (3H, *s*, CMeOH), 3.09 (1H, *s*, OH), 5.18 (1H, *s*, S—CH—S), 7.08–7.30 (4H, *m*, Ar), 7.43–7.52 (2H, *m*, Ar), 7.83 (1H, *d*, *J* = 8.4 Hz, Ar), 7.85 (1H, *d*, *J* = 8.4 Hz, Ar), 7.92–7.99 (4H, *m*, Ar). ^13^C NMR (50 MHz) δ (ppm): 20.0 (Me), 26.8 (CMe_3_), 38.6 (CMe_3_), 76.1 (COH), 79.4 (S—CH—S), 126.1, 126.2, 126.4, 127.4, 127.9, 128.0, 128.5 (Ar CH), 129.0 (Ar C), 129.1, 131.8 (Ar CH), 131.9, 132.0, 132.1 (Ar C), 132.8 (Ar CH), 133.5, 133.7, 141.8, 142.6 (Ar C).

## 6. Refinement

Crystal data, data collection and structure refinement details are summarized in Table 3 ▸. All H atoms were refined using a riding model with *d*(C—H) = 0.95 Å, *U*_iso_ = 1.2*U*_eq_ (C) for aromatic H, 1.00 Å, *U*_iso_ = 1.2*U*_eq_ (C) for CH, 0.98 Å, *U*_iso_ = 1.5*U*_eq_ (C) for methyl H atoms and *d*(O—H) = 0.84 Å, *U*_iso_ = 1.5*U*_eq_ (O) for OH.

## Supplementary Material

Crystal structure: contains datablock(s) 1, 2, global. DOI: 10.1107/S2056989023000476/hb8049sup1.cif

Structure factors: contains datablock(s) 1. DOI: 10.1107/S2056989023000476/hb80491sup2.hkl

Structure factors: contains datablock(s) 2. DOI: 10.1107/S2056989023000476/hb80492sup3.hkl

Supporting information file. DOI: 10.1107/S2056989023000476/hb80491sup4.cml

Supporting information file. DOI: 10.1107/S2056989023000476/hb80492sup5.cml

CCDC references: 2236820, 2236819

Additional supporting information:  crystallographic information; 3D view; checkCIF report

## Figures and Tables

**Table 1:** Hydrogen-bond geometry (Å, °) for **1** [Chem scheme1] *Cg*3 is the centroid of the C201–C204/C210/C209 ring.

*D*—H⋯*A*	*D*—H	H⋯*A*	*D*⋯*A*	*D*—H⋯*A*
O2—H2*O*⋯S2^i^	0.84	2.69	3.341 (2)	136
C105—H105⋯S1^ii^	0.95	2.90	3.580 (2)	130
C106—H106⋯*Cg*3^ii^	0.95	2.87	3.719 (3)	150

Symmetry codes: (i) 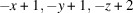 ; (ii) 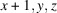 .

**Table 2:** Hydrogen-bond geometry (Å, °) for **2** [Chem scheme1] *Cg*2 and *Cg*4 are the centroids of the C105–C110 and C205–C210 rings, respectively.

*D*—H⋯*A*	*D*—H	H⋯*A*	*D*⋯*A*	*D*—H⋯*A*
O2—H2⋯S1	0.84	2.52	2.9942 (14)	117
C203—H203⋯*Cg*2^i^	0.95	2.60	3.4606 (19)	151
C103—H103⋯*Cg*4^ii^	0.95	2.93	3.443 (2)	115

Symmetry codes: (i) 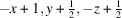 ; (ii) 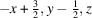 .

**Table 3:** Experimental details

	**1**	**2**
Crystal data		
Chemical formula	C_26_H_24_OS_2_	C_27_H_26_OS_2_
*M*_r_	416.57	430.60
Crystal system, space group	Triclinic, *P* 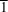	Orthorhombic, *P* *b* *c* *a*
Temperature (K)	163	163
*a*, *b*, *c* (Å)	9.322 (3), 11.064 (4), 11.207 (4)	17.565 (5), 11.103 (3), 22.977 (7)
α, β, γ (°)	81.607 (4), 84.444 (5), 69.411 (4)	90, 90, 90
*V* (Å^3^)	1069.2 (6)	4481 (2)
*Z*	2	8
Radiation type	Mo *K*α	Mo *K*α
μ (mm^−1^)	0.26	0.25
Crystal size (mm)	–	0.55 × 0.45 × 0.12

Data collection		
Diffractometer	Bruker SMART CCD	Bruker SMART CCD
Absorption correction	Multi-scan (*SADABS*; Krause *et al.*, 2015 ▸)	Multi-scan (*SADABS*; Krause *et al.*, 2015 ▸)
*T*_min_, *T*_max_	0.768, 1.000	0.822, 1.000
No. of measured, independent and observed [*I* > 2σ(*I*)] reflections	13453, 4290, 3838	48381, 4501, 3668
*R*_int_	0.021	0.038
(sin θ/λ)_max_ (Å^−1^)	0.626	0.625

Refinement		
*R*[*F* ^2^ > 2σ(*F* ^2^)], *wR*(*F* ^2^), *S*	0.047, 0.135, 1.07	0.031, 0.087, 1.05
No. of reflections	4290	4501
No. of parameters	266	276
H-atom treatment	H-atom parameters constrained	H-atom parameters constrained
Δρ_max_, Δρ_min_ (e Å^−3^)	1.16, −0.36	0.28, −0.24

Computer programs: *SMART* and *SAINT* (Bruker, 1998 ▸), *SHELXT* (Sheldrick, 2015*a* ▸), *Mercury* (Macrae *et al.*, 2008 ▸), *SHELXL2019/2* (Sheldrick, 2015*b* ▸) and *publCIF* (Westrip 2010 ▸).

## supplementary crystallographic information

### 1-(Dinaphtho[2,1-d:1',2'-f][1,3]dithiepin-4-yl)-2,2-dimethylpropan-1-ol (1) Crystal data

**Table:**

C_26_H_24_OS_2_	*Z* = 2
*M**_r_* = 416.57	*F*(000) = 440
Triclinic, *P*1	*D*_x_ = 1.294 Mg m^−^^3^
*a* = 9.322 (3) Å	Mo *K*α radiation, λ = 0.71073 Å
*b* = 11.064 (4) Å	Cell parameters from 4367 reflections
*c* = 11.207 (4) Å	θ = 2.9–26.4°
α = 81.607 (4)°	µ = 0.26 mm^−^^1^
β = 84.444 (5)°	*T* = 163 K
γ = 69.411 (4)°	Block, colourless
*V* = 1069.2 (6) Å^3^	

### 1-(Dinaphtho[2,1-d:1',2'-f][1,3]dithiepin-4-yl)-2,2-dimethylpropan-1-ol (1) Data collection

**Table:**

Bruker SMART CCD diffractometer	3838 reflections with *I* > 2σ(*I*)
Radiation source: sealed tube	*R*_int_ = 0.021
ω scans	θ_max_ = 26.4°, θ_min_ = 2.3°
Absorption correction: multi-scan (SADABS; Krause *et al.*, 2015)	*h* = −11→11
*T*_min_ = 0.768, *T*_max_ = 1.000	*k* = −13→13
13453 measured reflections	*l* = −10→13
4290 independent reflections	

### 1-(Dinaphtho[2,1-d:1',2'-f][1,3]dithiepin-4-yl)-2,2-dimethylpropan-1-ol (1) Refinement

**Table:**

Refinement on *F*^2^	Primary atom site location: dual
Least-squares matrix: full	Hydrogen site location: inferred from neighbouring sites
*R*[*F*^2^ > 2σ(*F*^2^)] = 0.047	H-atom parameters constrained
*wR*(*F*^2^) = 0.135	*w* = 1/[σ^2^(*F*_o_^2^) + (0.074*P*)^2^ + 0.8428*P*] where *P* = (*F*_o_^2^ + 2*F*_c_^2^)/3
*S* = 1.07	(Δ/σ)_max_ < 0.001
4290 reflections	Δρ_max_ = 1.16 e Å^−^^3^
266 parameters	Δρ_min_ = −0.36 e Å^−^^3^
0 restraints	

### 1-(Dinaphtho[2,1-d:1',2'-f][1,3]dithiepin-4-yl)-2,2-dimethylpropan-1-ol (1) Special details

**Table:**

Geometry. All esds (except the esd in the dihedral angle between two l.s. planes) are estimated using the full covariance matrix. The cell esds are taken into account individually in the estimation of esds in distances, angles and torsion angles; correlations between esds in cell parameters are only used when they are defined by crystal symmetry. An approximate (isotropic) treatment of cell esds is used for estimating esds involving l.s. planes.

### 1-(Dinaphtho[2,1-d:1',2'-f][1,3]dithiepin-4-yl)-2,2-dimethylpropan-1-ol (1) Fractional atomic coordinates and isotropic or equivalent isotropic displacement parameters (Å^2^)

**Table:**

	*x*	*y*	*z*	*U*_iso_*/*U*_eq_	
S1	0.33969 (6)	0.51235 (5)	0.66880 (4)	0.02624 (15)	
S2	0.42915 (6)	0.38952 (5)	0.92483 (5)	0.03187 (16)	
O2	0.3503 (2)	0.70274 (19)	0.83735 (18)	0.0523 (5)	
H2O	0.436483	0.656981	0.863736	0.079*	
C101	0.6401 (2)	0.35435 (19)	0.69959 (17)	0.0224 (4)	
C102	0.5416 (2)	0.47915 (19)	0.66592 (17)	0.0233 (4)	
C103	0.5973 (2)	0.5826 (2)	0.62546 (19)	0.0276 (4)	
H103	0.527808	0.667316	0.601632	0.033*	
C104	0.7515 (3)	0.5601 (2)	0.6209 (2)	0.0294 (4)	
H104	0.788381	0.629548	0.592874	0.035*	
C105	1.0173 (2)	0.4115 (2)	0.6538 (2)	0.0301 (5)	
H105	1.054619	0.480523	0.624993	0.036*	
C106	1.1189 (2)	0.2914 (2)	0.6913 (2)	0.0311 (5)	
H106	1.225710	0.277176	0.687807	0.037*	
C107	1.0640 (2)	0.1884 (2)	0.7353 (2)	0.0306 (5)	
H107	1.134365	0.105357	0.762955	0.037*	
C108	0.9104 (2)	0.2071 (2)	0.73834 (19)	0.0268 (4)	
H108	0.875883	0.136539	0.767596	0.032*	
C109	0.8016 (2)	0.33038 (19)	0.69854 (17)	0.0232 (4)	
C110	0.8571 (2)	0.4352 (2)	0.65708 (18)	0.0256 (4)	
C201	0.5782 (2)	0.24680 (19)	0.74177 (18)	0.0234 (4)	
C202	0.4836 (2)	0.2524 (2)	0.84438 (18)	0.0256 (4)	
C203	0.4292 (2)	0.1488 (2)	0.8892 (2)	0.0317 (5)	
H203	0.364920	0.154364	0.960880	0.038*	
C204	0.4684 (3)	0.0413 (2)	0.8304 (2)	0.0329 (5)	
H204	0.433658	−0.028498	0.862557	0.039*	
C205	0.5972 (3)	−0.0763 (2)	0.6582 (2)	0.0389 (6)	
H205	0.562432	−0.146393	0.689069	0.047*	
C206	0.6824 (3)	−0.0812 (2)	0.5528 (2)	0.0444 (6)	
H206	0.706735	−0.154604	0.510160	0.053*	
C207	0.7352 (3)	0.0215 (2)	0.5060 (2)	0.0396 (5)	
H207	0.794904	0.016518	0.432010	0.048*	
C208	0.7019 (3)	0.1285 (2)	0.5654 (2)	0.0301 (4)	
H208	0.737903	0.197228	0.532074	0.036*	
C209	0.6144 (2)	0.13735 (19)	0.67589 (18)	0.0249 (4)	
C210	0.5597 (2)	0.0330 (2)	0.7226 (2)	0.0293 (5)	
C1	0.2830 (3)	0.5109 (2)	0.8279 (2)	0.0352 (5)	
H1	0.189445	0.485240	0.839490	0.042*	
C2	0.2412 (3)	0.6405 (2)	0.8794 (2)	0.0418 (6)	
H2	0.248695	0.619635	0.968939	0.050*	
C3	0.0772 (3)	0.7391 (2)	0.8560 (2)	0.0416 (6)	
C4	0.0518 (3)	0.7846 (3)	0.7226 (3)	0.0542 (7)	
H4A	0.141715	0.803457	0.683974	0.081*	
H4B	−0.039111	0.863395	0.713553	0.081*	
H4C	0.036433	0.716157	0.684337	0.081*	
C5	0.0606 (5)	0.8572 (3)	0.9196 (3)	0.0774 (12)	
H5A	0.076028	0.829663	1.006016	0.116*	
H5B	−0.042288	0.921700	0.908727	0.116*	
H5C	0.137525	0.895767	0.884872	0.116*	
C6	−0.0428 (5)	0.6797 (4)	0.9093 (5)	0.0960 (16)	
H6A	−0.020465	0.642462	0.993122	0.144*	
H6B	−0.040525	0.611050	0.862058	0.144*	
H6C	−0.144613	0.747157	0.906937	0.144*	

### 1-(Dinaphtho[2,1-d:1',2'-f][1,3]dithiepin-4-yl)-2,2-dimethylpropan-1-ol (1) Atomic displacement parameters (Å^2^)

**Table:**

	*U*^11^	*U*^22^	*U*^33^	*U*^12^	*U*^13^	*U*^23^
S1	0.0222 (3)	0.0305 (3)	0.0242 (3)	−0.0072 (2)	−0.00247 (18)	−0.00161 (19)
S2	0.0381 (3)	0.0304 (3)	0.0227 (3)	−0.0063 (2)	−0.0030 (2)	−0.0022 (2)
O2	0.0655 (13)	0.0478 (11)	0.0519 (11)	−0.0269 (10)	−0.0113 (10)	−0.0072 (9)
C101	0.0255 (10)	0.0240 (9)	0.0205 (9)	−0.0117 (8)	−0.0009 (7)	−0.0034 (7)
C102	0.0243 (9)	0.0248 (10)	0.0219 (9)	−0.0093 (8)	−0.0032 (7)	−0.0025 (7)
C103	0.0310 (11)	0.0215 (9)	0.0286 (10)	−0.0077 (8)	−0.0038 (8)	−0.0002 (8)
C104	0.0338 (11)	0.0245 (10)	0.0332 (11)	−0.0153 (9)	−0.0016 (9)	0.0003 (8)
C105	0.0305 (11)	0.0337 (11)	0.0322 (11)	−0.0190 (9)	−0.0010 (8)	−0.0030 (9)
C106	0.0229 (10)	0.0404 (12)	0.0330 (11)	−0.0142 (9)	−0.0021 (8)	−0.0054 (9)
C107	0.0269 (10)	0.0299 (11)	0.0325 (11)	−0.0068 (9)	−0.0043 (8)	−0.0019 (9)
C108	0.0272 (10)	0.0248 (10)	0.0286 (10)	−0.0099 (8)	−0.0029 (8)	−0.0009 (8)
C109	0.0256 (10)	0.0236 (9)	0.0226 (9)	−0.0109 (8)	−0.0018 (7)	−0.0026 (7)
C110	0.0285 (10)	0.0271 (10)	0.0248 (10)	−0.0141 (8)	−0.0015 (8)	−0.0028 (8)
C201	0.0217 (9)	0.0209 (9)	0.0277 (10)	−0.0082 (7)	−0.0055 (8)	0.0014 (7)
C202	0.0257 (10)	0.0254 (10)	0.0249 (10)	−0.0081 (8)	−0.0048 (8)	0.0004 (8)
C203	0.0276 (11)	0.0344 (11)	0.0310 (11)	−0.0126 (9)	−0.0024 (8)	0.0079 (9)
C204	0.0313 (11)	0.0293 (11)	0.0401 (12)	−0.0166 (9)	−0.0091 (9)	0.0100 (9)
C205	0.0515 (14)	0.0229 (11)	0.0468 (14)	−0.0168 (10)	−0.0180 (11)	0.0024 (9)
C206	0.0649 (17)	0.0241 (11)	0.0440 (14)	−0.0108 (11)	−0.0134 (12)	−0.0080 (10)
C207	0.0503 (14)	0.0320 (12)	0.0343 (12)	−0.0094 (11)	−0.0039 (10)	−0.0071 (9)
C208	0.0337 (11)	0.0262 (10)	0.0305 (11)	−0.0103 (9)	−0.0041 (9)	−0.0021 (8)
C209	0.0247 (10)	0.0225 (9)	0.0286 (10)	−0.0094 (8)	−0.0070 (8)	0.0005 (8)
C210	0.0317 (11)	0.0233 (10)	0.0343 (11)	−0.0111 (8)	−0.0125 (9)	0.0042 (8)
C1	0.0378 (12)	0.0341 (12)	0.0260 (11)	−0.0048 (10)	0.0001 (9)	0.0007 (9)
C2	0.0597 (16)	0.0358 (13)	0.0271 (11)	−0.0129 (11)	−0.0022 (10)	−0.0038 (9)
C3	0.0484 (14)	0.0281 (11)	0.0383 (13)	−0.0040 (10)	0.0106 (11)	−0.0036 (9)
C4	0.0498 (16)	0.0470 (15)	0.0469 (15)	0.0086 (13)	−0.0090 (12)	−0.0055 (12)
C5	0.127 (3)	0.0404 (16)	0.0465 (17)	−0.0064 (18)	0.0000 (19)	−0.0079 (13)
C6	0.065 (2)	0.065 (2)	0.136 (4)	−0.0105 (19)	0.047 (2)	−0.006 (2)

### 1-(Dinaphtho[2,1-d:1',2'-f][1,3]dithiepin-4-yl)-2,2-dimethylpropan-1-ol (1) Geometric parameters (Å, º)

**Table:**

S1—C102	1.784 (2)	C204—C210	1.404 (3)
S1—C1	1.809 (2)	C204—H204	0.9500
S2—C202	1.772 (2)	C205—C206	1.355 (4)
S2—C1	1.848 (2)	C205—C210	1.419 (3)
O2—C2	1.426 (3)	C205—H205	0.9500
O2—H2O	0.8400	C206—C207	1.406 (4)
C101—C102	1.384 (3)	C206—H206	0.9500
C101—C109	1.433 (3)	C207—C208	1.368 (3)
C101—C201	1.496 (3)	C207—H207	0.9500
C102—C103	1.417 (3)	C208—C209	1.411 (3)
C103—C104	1.367 (3)	C208—H208	0.9500
C103—H103	0.9500	C209—C210	1.433 (3)
C104—C110	1.414 (3)	C1—C2	1.532 (3)
C104—H104	0.9500	C1—H1	1.0000
C105—C106	1.366 (3)	C2—C3	1.555 (4)
C105—C110	1.421 (3)	C2—H2	1.0000
C105—H105	0.9500	C3—C4	1.520 (4)
C106—C107	1.414 (3)	C3—C6	1.521 (5)
C106—H106	0.9500	C3—C5	1.533 (4)
C107—C108	1.371 (3)	C4—H4A	0.9800
C107—H107	0.9500	C4—H4B	0.9800
C108—C109	1.423 (3)	C4—H4C	0.9800
C108—H108	0.9500	C5—H5A	0.9800
C109—C110	1.430 (3)	C5—H5B	0.9800
C201—C202	1.375 (3)	C5—H5C	0.9800
C201—C209	1.432 (3)	C6—H6A	0.9800
C202—C203	1.420 (3)	C6—H6B	0.9800
C203—C204	1.364 (3)	C6—H6C	0.9800
C203—H203	0.9500		
			
C102—S1—C1	103.57 (10)	C207—C206—H206	119.7
C202—S2—C1	101.51 (10)	C208—C207—C206	121.0 (2)
C2—O2—H2O	109.5	C208—C207—H207	119.5
C102—C101—C109	119.27 (18)	C206—C207—H207	119.5
C102—C101—C201	120.37 (18)	C207—C208—C209	120.4 (2)
C109—C101—C201	120.31 (17)	C207—C208—H208	119.8
C101—C102—C103	121.48 (19)	C209—C208—H208	119.8
C101—C102—S1	120.23 (15)	C208—C209—C201	122.31 (18)
C103—C102—S1	118.26 (15)	C208—C209—C210	118.36 (19)
C104—C103—C102	119.70 (19)	C201—C209—C210	119.33 (19)
C104—C103—H103	120.1	C204—C210—C205	121.1 (2)
C102—C103—H103	120.1	C204—C210—C209	119.5 (2)
C103—C104—C110	121.20 (19)	C205—C210—C209	119.4 (2)
C103—C104—H104	119.4	C2—C1—S1	116.18 (16)
C110—C104—H104	119.4	C2—C1—S2	106.60 (16)
C106—C105—C110	121.38 (19)	S1—C1—S2	113.04 (12)
C106—C105—H105	119.3	C2—C1—H1	106.8
C110—C105—H105	119.3	S1—C1—H1	106.8
C105—C106—C107	119.6 (2)	S2—C1—H1	106.8
C105—C106—H106	120.2	O2—C2—C1	110.7 (2)
C107—C106—H106	120.2	O2—C2—C3	108.9 (2)
C108—C107—C106	120.7 (2)	C1—C2—C3	116.1 (2)
C108—C107—H107	119.7	O2—C2—H2	106.9
C106—C107—H107	119.7	C1—C2—H2	106.9
C107—C108—C109	121.21 (19)	C3—C2—H2	106.9
C107—C108—H108	119.4	C4—C3—C6	109.3 (3)
C109—C108—H108	119.4	C4—C3—C5	108.4 (2)
C108—C109—C110	117.97 (18)	C6—C3—C5	109.5 (3)
C108—C109—C101	123.16 (18)	C4—C3—C2	112.7 (2)
C110—C109—C101	118.87 (18)	C6—C3—C2	110.3 (2)
C104—C110—C105	121.46 (18)	C5—C3—C2	106.5 (3)
C104—C110—C109	119.42 (19)	C3—C4—H4A	109.5
C105—C110—C109	119.13 (19)	C3—C4—H4B	109.5
C202—C201—C209	118.82 (18)	H4A—C4—H4B	109.5
C202—C201—C101	120.21 (18)	C3—C4—H4C	109.5
C209—C201—C101	120.97 (18)	H4A—C4—H4C	109.5
C201—C202—C203	121.20 (19)	H4B—C4—H4C	109.5
C201—C202—S2	120.23 (16)	C3—C5—H5A	109.5
C203—C202—S2	118.56 (16)	C3—C5—H5B	109.5
C204—C203—C202	120.6 (2)	H5A—C5—H5B	109.5
C204—C203—H203	119.7	C3—C5—H5C	109.5
C202—C203—H203	119.7	H5A—C5—H5C	109.5
C203—C204—C210	120.46 (19)	H5B—C5—H5C	109.5
C203—C204—H204	119.8	C3—C6—H6A	109.5
C210—C204—H204	119.8	C3—C6—H6B	109.5
C206—C205—C210	120.3 (2)	H6A—C6—H6B	109.5
C206—C205—H205	119.9	C3—C6—H6C	109.5
C210—C205—H205	119.9	H6A—C6—H6C	109.5
C205—C206—C207	120.6 (2)	H6B—C6—H6C	109.5
C205—C206—H206	119.7		
			
C109—C101—C102—C103	2.8 (3)	C1—S2—C202—C203	−104.56 (18)
C201—C101—C102—C103	−179.89 (18)	C201—C202—C203—C204	−0.7 (3)
C109—C101—C102—S1	−178.94 (14)	S2—C202—C203—C204	−179.89 (16)
C201—C101—C102—S1	−1.6 (3)	C202—C203—C204—C210	−1.8 (3)
C1—S1—C102—C101	75.24 (18)	C210—C205—C206—C207	0.2 (4)
C1—S1—C102—C103	−106.41 (17)	C205—C206—C207—C208	0.0 (4)
C101—C102—C103—C104	−0.9 (3)	C206—C207—C208—C209	0.5 (4)
S1—C102—C103—C104	−179.27 (16)	C207—C208—C209—C201	179.4 (2)
C102—C103—C104—C110	−0.7 (3)	C207—C208—C209—C210	−1.1 (3)
C110—C105—C106—C107	−0.5 (3)	C202—C201—C209—C208	175.57 (19)
C105—C106—C107—C108	1.4 (3)	C101—C201—C209—C208	−3.7 (3)
C106—C107—C108—C109	−0.5 (3)	C202—C201—C209—C210	−4.0 (3)
C107—C108—C109—C110	−1.1 (3)	C101—C201—C209—C210	176.66 (17)
C107—C108—C109—C101	179.71 (19)	C203—C204—C210—C205	−177.9 (2)
C102—C101—C109—C108	176.21 (18)	C203—C204—C210—C209	1.3 (3)
C201—C101—C109—C108	−1.1 (3)	C206—C205—C210—C204	178.4 (2)
C102—C101—C109—C110	−3.0 (3)	C206—C205—C210—C209	−0.8 (3)
C201—C101—C109—C110	179.67 (17)	C208—C209—C210—C204	−178.01 (19)
C103—C104—C110—C105	−179.3 (2)	C201—C209—C210—C204	1.6 (3)
C103—C104—C110—C109	0.4 (3)	C208—C209—C210—C205	1.2 (3)
C106—C105—C110—C104	178.6 (2)	C201—C209—C210—C205	−179.20 (18)
C106—C105—C110—C109	−1.1 (3)	C102—S1—C1—C2	88.26 (19)
C108—C109—C110—C104	−177.79 (19)	C102—S1—C1—S2	−35.46 (16)
C101—C109—C110—C104	1.4 (3)	C202—S2—C1—C2	−172.79 (16)
C108—C109—C110—C105	1.9 (3)	C202—S2—C1—S1	−43.95 (16)
C101—C109—C110—C105	−178.90 (18)	S1—C1—C2—O2	−44.5 (3)
C102—C101—C201—C202	−62.5 (3)	S2—C1—C2—O2	82.5 (2)
C109—C101—C201—C202	114.9 (2)	S1—C1—C2—C3	80.3 (2)
C102—C101—C201—C209	116.8 (2)	S2—C1—C2—C3	−152.75 (18)
C109—C101—C201—C209	−65.8 (2)	O2—C2—C3—C4	62.2 (3)
C209—C201—C202—C203	3.6 (3)	C1—C2—C3—C4	−63.5 (3)
C101—C201—C202—C203	−177.04 (18)	O2—C2—C3—C6	−175.2 (3)
C209—C201—C202—S2	−177.24 (14)	C1—C2—C3—C6	59.0 (3)
C101—C201—C202—S2	2.1 (3)	O2—C2—C3—C5	−56.4 (3)
C1—S2—C202—C201	76.29 (18)	C1—C2—C3—C5	177.8 (2)

### 1-(Dinaphtho[2,1-d:1',2'-f][1,3]dithiepin-4-yl)-2,2-dimethylpropan-1-ol (1) Hydrogen-bond geometry (Å, º)

*Cg*3 is the centroid of the C201–C204/C210/C209 ring.

**Table:**

*D*—H···*A*	*D*—H	H···*A*	*D*···*A*	*D*—H···*A*
O2—H2*O*···S2^i^	0.84	2.69	3.341 (2)	136
C105—H105···S1^ii^	0.95	2.90	3.580 (2)	130
C106—H106···*Cg*3^ii^	0.95	2.87	3.719 (3)	150

Symmetry codes: (i) −*x*+1, −*y*+1, −*z*+2; (ii) *x*+1, *y*, *z*.

### 2-(Dinaphtho[2,1-d:1',2'-f][1,3]dithiepin-4-yl)-3,3-dimethylbutan-2-ol (2) Crystal data

**Table:**

C_27_H_26_OS_2_	*D*_x_ = 1.277 Mg m^−^^3^
*M**_r_* = 430.60	Mo *K*α radiation, λ = 0.71073 Å
Orthorhombic, *P**b**c**a*	Cell parameters from 6104 reflections
*a* = 17.565 (5) Å	θ = 2.9–26.3°
*b* = 11.103 (3) Å	µ = 0.25 mm^−^^1^
*c* = 22.977 (7) Å	*T* = 163 K
*V* = 4481 (2) Å^3^	Block, colourless
*Z* = 8	0.55 × 0.45 × 0.12 mm
*F*(000) = 1824	

### 2-(Dinaphtho[2,1-d:1',2'-f][1,3]dithiepin-4-yl)-3,3-dimethylbutan-2-ol (2) Data collection

**Table:**

Bruker SMART CCD diffractometer	3668 reflections with *I* > 2σ(*I*)
Radiation source: sealed tube	*R*_int_ = 0.038
ω scans	θ_max_ = 26.4°, θ_min_ = 2.1°
Absorption correction: multi-scan (SADABS; Krause *et al.*, 2015)	*h* = −21→21
*T*_min_ = 0.822, *T*_max_ = 1.000	*k* = −13→7
48381 measured reflections	*l* = −28→28
4501 independent reflections	

### 2-(Dinaphtho[2,1-d:1',2'-f][1,3]dithiepin-4-yl)-3,3-dimethylbutan-2-ol (2) Refinement

**Table:**

Refinement on *F*^2^	Primary atom site location: dual
Least-squares matrix: full	Hydrogen site location: inferred from neighbouring sites
*R*[*F*^2^ > 2σ(*F*^2^)] = 0.031	H-atom parameters constrained
*wR*(*F*^2^) = 0.087	*w* = 1/[σ^2^(*F*_o_^2^) + (0.0489*P*)^2^ + 0.9358*P*] where *P* = (*F*_o_^2^ + 2*F*_c_^2^)/3
*S* = 1.05	(Δ/σ)_max_ = 0.001
4501 reflections	Δρ_max_ = 0.28 e Å^−^^3^
276 parameters	Δρ_min_ = −0.24 e Å^−^^3^
0 restraints	

### 2-(Dinaphtho[2,1-d:1',2'-f][1,3]dithiepin-4-yl)-3,3-dimethylbutan-2-ol (2) Special details

**Table:**

Geometry. All esds (except the esd in the dihedral angle between two l.s. planes) are estimated using the full covariance matrix. The cell esds are taken into account individually in the estimation of esds in distances, angles and torsion angles; correlations between esds in cell parameters are only used when they are defined by crystal symmetry. An approximate (isotropic) treatment of cell esds is used for estimating esds involving l.s. planes.

### 2-(Dinaphtho[2,1-d:1',2'-f][1,3]dithiepin-4-yl)-3,3-dimethylbutan-2-ol (2) Fractional atomic coordinates and isotropic or equivalent isotropic displacement parameters (Å^2^)

**Table:**

	*x*	*y*	*z*	*U*_iso_*/*U*_eq_	
S1	0.73428 (2)	0.32278 (3)	0.25341 (2)	0.02829 (11)	
S2	0.57569 (2)	0.35112 (3)	0.30806 (2)	0.02663 (10)	
O2	0.77271 (6)	0.20287 (13)	0.36637 (5)	0.0479 (3)	
H2	0.792870	0.197942	0.333304	0.072*	
C101	0.61144 (8)	0.32916 (12)	0.17891 (6)	0.0239 (3)	
C102	0.67252 (8)	0.26247 (12)	0.19914 (6)	0.0259 (3)	
C103	0.68946 (9)	0.14850 (13)	0.17479 (7)	0.0319 (3)	
H103	0.732573	0.104691	0.188011	0.038*	
C104	0.64385 (9)	0.10128 (13)	0.13222 (6)	0.0341 (4)	
H104	0.657238	0.026560	0.114873	0.041*	
C105	0.52481 (10)	0.10881 (15)	0.07366 (6)	0.0380 (4)	
H105	0.535790	0.031949	0.057546	0.046*	
C106	0.45920 (11)	0.16638 (16)	0.05810 (7)	0.0425 (4)	
H106	0.424837	0.129142	0.031728	0.051*	
C107	0.44247 (10)	0.28053 (16)	0.08105 (7)	0.0396 (4)	
H107	0.396725	0.320332	0.070249	0.048*	
C108	0.49224 (9)	0.33496 (13)	0.11920 (6)	0.0312 (3)	
H108	0.480660	0.412796	0.133911	0.037*	
C109	0.56037 (8)	0.27724 (12)	0.13696 (6)	0.0264 (3)	
C110	0.57705 (9)	0.16155 (13)	0.11349 (6)	0.0302 (3)	
C201	0.59921 (8)	0.45406 (12)	0.20081 (6)	0.0232 (3)	
C202	0.58292 (8)	0.47451 (12)	0.25909 (6)	0.0237 (3)	
C203	0.57257 (8)	0.59249 (13)	0.28098 (6)	0.0259 (3)	
H203	0.560058	0.604415	0.320791	0.031*	
C204	0.58057 (8)	0.68877 (13)	0.24491 (6)	0.0266 (3)	
H204	0.572416	0.767657	0.259657	0.032*	
C205	0.61547 (9)	0.77265 (13)	0.14852 (6)	0.0302 (3)	
H205	0.608553	0.852015	0.163038	0.036*	
C206	0.63931 (9)	0.75659 (14)	0.09223 (7)	0.0350 (4)	
H206	0.649514	0.824584	0.068372	0.042*	
C207	0.64870 (9)	0.63969 (14)	0.06970 (6)	0.0351 (4)	
H207	0.665319	0.628986	0.030693	0.042*	
C208	0.63402 (9)	0.54164 (13)	0.10370 (6)	0.0295 (3)	
H208	0.639712	0.463207	0.087764	0.035*	
C209	0.61029 (8)	0.55475 (12)	0.16274 (6)	0.0236 (3)	
C210	0.60099 (8)	0.67326 (12)	0.18533 (6)	0.0242 (3)	
C1	0.67673 (8)	0.32372 (13)	0.32134 (6)	0.0260 (3)	
H1	0.695201	0.394259	0.344427	0.031*	
C2	0.69130 (9)	0.21063 (13)	0.36018 (6)	0.0311 (3)	
C22	0.66153 (11)	0.09668 (14)	0.33065 (7)	0.0441 (4)	
H22A	0.668752	0.027603	0.356614	0.066*	
H22B	0.689526	0.083149	0.294336	0.066*	
H22C	0.607217	0.106213	0.322042	0.066*	
C3	0.66090 (9)	0.22442 (14)	0.42475 (6)	0.0341 (4)	
C4	0.70379 (12)	0.1358 (2)	0.46484 (8)	0.0589 (6)	
H4A	0.694488	0.053034	0.451776	0.088*	
H4B	0.685593	0.145161	0.504909	0.088*	
H4C	0.758489	0.152811	0.463292	0.088*	
C5	0.57532 (10)	0.19676 (16)	0.43056 (8)	0.0440 (4)	
H5A	0.565400	0.114255	0.417450	0.066*	
H5B	0.546176	0.253339	0.406580	0.066*	
H5C	0.559937	0.205096	0.471365	0.066*	
C6	0.67636 (12)	0.35085 (16)	0.44817 (7)	0.0494 (5)	
H6A	0.730208	0.370831	0.442579	0.074*	
H6B	0.664061	0.353651	0.489751	0.074*	
H6C	0.644719	0.409185	0.427202	0.074*	

### 2-(Dinaphtho[2,1-d:1',2'-f][1,3]dithiepin-4-yl)-3,3-dimethylbutan-2-ol (2) Atomic displacement parameters (Å^2^)

**Table:**

	*U*^11^	*U*^22^	*U*^33^	*U*^12^	*U*^13^	*U*^23^
S1	0.02315 (19)	0.0351 (2)	0.02660 (19)	−0.00032 (14)	0.00222 (14)	0.00127 (15)
S2	0.02381 (19)	0.0310 (2)	0.02514 (18)	0.00074 (14)	0.00317 (14)	0.00552 (14)
O2	0.0308 (6)	0.0716 (9)	0.0413 (7)	0.0141 (6)	0.0026 (5)	0.0183 (6)
C101	0.0277 (7)	0.0227 (7)	0.0211 (7)	−0.0025 (6)	0.0044 (5)	0.0002 (5)
C102	0.0263 (7)	0.0260 (7)	0.0252 (7)	−0.0010 (6)	0.0048 (6)	0.0003 (5)
C103	0.0336 (8)	0.0283 (8)	0.0338 (8)	0.0048 (6)	0.0081 (7)	0.0012 (6)
C104	0.0469 (9)	0.0223 (7)	0.0333 (8)	−0.0022 (7)	0.0136 (7)	−0.0051 (6)
C105	0.0553 (11)	0.0351 (8)	0.0235 (7)	−0.0155 (8)	0.0077 (7)	−0.0064 (6)
C106	0.0531 (11)	0.0492 (10)	0.0251 (8)	−0.0211 (9)	−0.0044 (8)	−0.0023 (7)
C107	0.0408 (9)	0.0494 (10)	0.0286 (8)	−0.0089 (8)	−0.0053 (7)	0.0062 (7)
C108	0.0356 (8)	0.0328 (8)	0.0252 (7)	−0.0039 (6)	−0.0007 (6)	0.0029 (6)
C109	0.0335 (8)	0.0264 (7)	0.0193 (7)	−0.0053 (6)	0.0041 (6)	0.0013 (5)
C110	0.0408 (9)	0.0266 (7)	0.0233 (7)	−0.0086 (6)	0.0088 (6)	−0.0018 (6)
C201	0.0220 (7)	0.0244 (7)	0.0232 (7)	0.0004 (5)	−0.0013 (5)	−0.0015 (5)
C202	0.0215 (7)	0.0261 (7)	0.0234 (7)	0.0010 (5)	0.0008 (5)	0.0021 (5)
C203	0.0254 (7)	0.0309 (7)	0.0214 (7)	0.0035 (6)	0.0009 (6)	−0.0039 (6)
C204	0.0262 (7)	0.0251 (7)	0.0285 (7)	0.0035 (6)	−0.0002 (6)	−0.0055 (6)
C205	0.0343 (8)	0.0238 (7)	0.0324 (8)	0.0012 (6)	−0.0017 (6)	0.0004 (6)
C206	0.0454 (9)	0.0278 (8)	0.0317 (8)	−0.0019 (7)	0.0017 (7)	0.0072 (6)
C207	0.0454 (9)	0.0382 (9)	0.0218 (7)	−0.0026 (7)	0.0040 (7)	0.0017 (6)
C208	0.0362 (8)	0.0279 (7)	0.0244 (7)	−0.0011 (6)	0.0012 (6)	−0.0031 (6)
C209	0.0236 (7)	0.0253 (7)	0.0220 (7)	0.0003 (5)	−0.0006 (5)	−0.0010 (5)
C210	0.0218 (7)	0.0251 (7)	0.0256 (7)	0.0014 (5)	−0.0018 (6)	−0.0007 (5)
C1	0.0232 (7)	0.0304 (7)	0.0243 (7)	0.0006 (6)	0.0019 (6)	0.0009 (6)
C2	0.0288 (8)	0.0339 (8)	0.0306 (8)	0.0052 (6)	0.0022 (6)	0.0064 (6)
C22	0.0646 (12)	0.0270 (8)	0.0406 (9)	0.0041 (8)	0.0093 (8)	0.0027 (7)
C3	0.0383 (9)	0.0374 (9)	0.0266 (8)	0.0030 (7)	0.0017 (6)	0.0085 (6)
C4	0.0635 (13)	0.0740 (14)	0.0392 (10)	0.0173 (11)	0.0035 (9)	0.0263 (10)
C5	0.0441 (10)	0.0500 (10)	0.0378 (9)	−0.0037 (8)	0.0113 (8)	0.0070 (8)
C6	0.0676 (13)	0.0534 (11)	0.0272 (8)	−0.0114 (9)	0.0007 (8)	−0.0017 (7)

### 2-(Dinaphtho[2,1-d:1',2'-f][1,3]dithiepin-4-yl)-3,3-dimethylbutan-2-ol (2) Geometric parameters (Å, º)

**Table:**

S1—C102	1.7832 (15)	C204—H204	0.9500
S1—C1	1.8596 (15)	C205—C206	1.371 (2)
S2—C202	1.7773 (14)	C205—C210	1.413 (2)
S2—C1	1.8265 (15)	C205—H205	0.9500
O2—C2	1.4396 (19)	C206—C207	1.407 (2)
O2—H2	0.8400	C206—H206	0.9500
C101—C102	1.384 (2)	C207—C208	1.364 (2)
C101—C109	1.437 (2)	C207—H207	0.9500
C101—C201	1.4908 (19)	C208—C209	1.4265 (19)
C102—C103	1.415 (2)	C208—H208	0.9500
C103—C104	1.369 (2)	C209—C210	1.4239 (19)
C103—H103	0.9500	C1—C2	1.5616 (19)
C104—C110	1.418 (2)	C1—H1	1.0000
C104—H104	0.9500	C2—C22	1.528 (2)
C105—C106	1.366 (3)	C2—C3	1.584 (2)
C105—C110	1.422 (2)	C22—H22A	0.9800
C105—H105	0.9500	C22—H22B	0.9800
C106—C107	1.404 (2)	C22—H22C	0.9800
C106—H106	0.9500	C3—C6	1.528 (2)
C107—C108	1.378 (2)	C3—C5	1.540 (2)
C107—H107	0.9500	C3—C4	1.544 (2)
C108—C109	1.418 (2)	C4—H4A	0.9800
C108—H108	0.9500	C4—H4B	0.9800
C109—C110	1.424 (2)	C4—H4C	0.9800
C201—C202	1.3881 (19)	C5—H5A	0.9800
C201—C209	1.4327 (19)	C5—H5B	0.9800
C202—C203	1.4149 (19)	C5—H5C	0.9800
C203—C204	1.360 (2)	C6—H6A	0.9800
C203—H203	0.9500	C6—H6B	0.9800
C204—C210	1.426 (2)	C6—H6C	0.9800
			
C102—S1—C1	104.97 (7)	C206—C207—H207	119.9
C202—S2—C1	99.47 (6)	C207—C208—C209	121.22 (13)
C2—O2—H2	109.5	C207—C208—H208	119.4
C102—C101—C109	119.64 (13)	C209—C208—H208	119.4
C102—C101—C201	119.74 (13)	C210—C209—C208	118.32 (12)
C109—C101—C201	120.62 (13)	C210—C209—C201	118.88 (12)
C101—C102—C103	120.57 (14)	C208—C209—C201	122.72 (12)
C101—C102—S1	120.37 (11)	C205—C210—C209	118.86 (13)
C103—C102—S1	118.97 (11)	C205—C210—C204	121.71 (13)
C104—C103—C102	120.13 (15)	C209—C210—C204	119.38 (12)
C104—C103—H103	119.9	C2—C1—S2	112.86 (10)
C102—C103—H103	119.9	C2—C1—S1	112.71 (10)
C103—C104—C110	121.37 (14)	S2—C1—S1	112.89 (8)
C103—C104—H104	119.3	C2—C1—H1	105.9
C110—C104—H104	119.3	S2—C1—H1	105.9
C106—C105—C110	121.35 (15)	S1—C1—H1	105.9
C106—C105—H105	119.3	O2—C2—C22	109.52 (13)
C110—C105—H105	119.3	O2—C2—C1	105.51 (12)
C105—C106—C107	120.06 (15)	C22—C2—C1	110.85 (13)
C105—C106—H106	120.0	O2—C2—C3	104.36 (12)
C107—C106—H106	120.0	C22—C2—C3	112.37 (13)
C108—C107—C106	120.15 (16)	C1—C2—C3	113.72 (12)
C108—C107—H107	119.9	C2—C22—H22A	109.5
C106—C107—H107	119.9	C2—C22—H22B	109.5
C107—C108—C109	121.37 (15)	H22A—C22—H22B	109.5
C107—C108—H108	119.3	C2—C22—H22C	109.5
C109—C108—H108	119.3	H22A—C22—H22C	109.5
C108—C109—C110	118.20 (13)	H22B—C22—H22C	109.5
C108—C109—C101	122.59 (13)	C6—C3—C5	109.03 (14)
C110—C109—C101	119.17 (14)	C6—C3—C4	106.79 (15)
C104—C110—C105	122.35 (14)	C5—C3—C4	107.29 (14)
C104—C110—C109	118.76 (14)	C6—C3—C2	111.02 (13)
C105—C110—C109	118.85 (15)	C5—C3—C2	113.01 (13)
C202—C201—C209	119.30 (12)	C4—C3—C2	109.43 (13)
C202—C201—C101	120.49 (12)	C3—C4—H4A	109.5
C209—C201—C101	120.02 (12)	C3—C4—H4B	109.5
C201—C202—C203	121.39 (12)	H4A—C4—H4B	109.5
C201—C202—S2	119.96 (11)	C3—C4—H4C	109.5
C203—C202—S2	118.64 (10)	H4A—C4—H4C	109.5
C204—C203—C202	119.86 (13)	H4B—C4—H4C	109.5
C204—C203—H203	120.1	C3—C5—H5A	109.5
C202—C203—H203	120.1	C3—C5—H5B	109.5
C203—C204—C210	121.08 (13)	H5A—C5—H5B	109.5
C203—C204—H204	119.5	C3—C5—H5C	109.5
C210—C204—H204	119.5	H5A—C5—H5C	109.5
C206—C205—C210	121.19 (13)	H5B—C5—H5C	109.5
C206—C205—H205	119.4	C3—C6—H6A	109.5
C210—C205—H205	119.4	C3—C6—H6B	109.5
C205—C206—C207	120.19 (14)	H6A—C6—H6B	109.5
C205—C206—H206	119.9	C3—C6—H6C	109.5
C207—C206—H206	119.9	H6A—C6—H6C	109.5
C208—C207—C206	120.20 (14)	H6B—C6—H6C	109.5
C208—C207—H207	119.9		
			
C109—C101—C102—C103	6.2 (2)	C202—C203—C204—C210	−1.4 (2)
C201—C101—C102—C103	−173.77 (12)	C210—C205—C206—C207	1.0 (2)
C109—C101—C102—S1	−177.39 (10)	C205—C206—C207—C208	0.1 (2)
C201—C101—C102—S1	2.59 (18)	C206—C207—C208—C209	−1.1 (2)
C1—S1—C102—C101	70.88 (12)	C207—C208—C209—C210	1.0 (2)
C1—S1—C102—C103	−112.69 (12)	C207—C208—C209—C201	−175.80 (14)
C101—C102—C103—C104	−2.2 (2)	C202—C201—C209—C210	−2.7 (2)
S1—C102—C103—C104	−178.67 (11)	C101—C201—C209—C210	−177.69 (13)
C102—C103—C104—C110	−3.1 (2)	C202—C201—C209—C208	174.08 (13)
C110—C105—C106—C107	0.6 (2)	C101—C201—C209—C208	−0.9 (2)
C105—C106—C107—C108	0.2 (2)	C206—C205—C210—C209	−1.1 (2)
C106—C107—C108—C109	−1.0 (2)	C206—C205—C210—C204	176.20 (14)
C107—C108—C109—C110	1.1 (2)	C208—C209—C210—C205	0.1 (2)
C107—C108—C109—C101	−176.80 (13)	C201—C209—C210—C205	177.02 (13)
C102—C101—C109—C108	172.88 (13)	C208—C209—C210—C204	−177.26 (13)
C201—C101—C109—C108	−7.1 (2)	C201—C209—C210—C204	−0.3 (2)
C102—C101—C109—C110	−4.99 (19)	C203—C204—C210—C205	−174.84 (13)
C201—C101—C109—C110	175.03 (12)	C203—C204—C210—C209	2.4 (2)
C103—C104—C110—C105	−173.53 (14)	C202—S2—C1—C2	−175.38 (10)
C103—C104—C110—C109	4.2 (2)	C202—S2—C1—S1	−46.11 (9)
C106—C105—C110—C104	177.19 (14)	C102—S1—C1—C2	96.29 (11)
C106—C105—C110—C109	−0.6 (2)	C102—S1—C1—S2	−33.06 (9)
C108—C109—C110—C104	−178.13 (13)	S2—C1—C2—O2	−179.74 (10)
C101—C109—C110—C104	−0.16 (19)	S1—C1—C2—O2	50.90 (14)
C108—C109—C110—C105	−0.3 (2)	S2—C1—C2—C22	61.78 (15)
C101—C109—C110—C105	177.67 (13)	S1—C1—C2—C22	−67.57 (15)
C102—C101—C201—C202	−63.95 (19)	S2—C1—C2—C3	−65.97 (15)
C109—C101—C201—C202	116.03 (15)	S1—C1—C2—C3	164.68 (10)
C102—C101—C201—C209	110.97 (15)	O2—C2—C3—C6	74.26 (16)
C109—C101—C201—C209	−69.05 (17)	C22—C2—C3—C6	−167.15 (15)
C209—C201—C202—C203	3.8 (2)	C1—C2—C3—C6	−40.19 (18)
C101—C201—C202—C203	178.76 (13)	O2—C2—C3—C5	−162.86 (13)
C209—C201—C202—S2	−175.52 (10)	C22—C2—C3—C5	−44.27 (18)
C101—C201—C202—S2	−0.57 (18)	C1—C2—C3—C5	82.69 (16)
C1—S2—C202—C201	79.99 (12)	O2—C2—C3—C4	−43.37 (18)
C1—S2—C202—C203	−99.36 (12)	C22—C2—C3—C4	75.21 (18)
C201—C202—C203—C204	−1.8 (2)	C1—C2—C3—C4	−157.83 (14)
S2—C202—C203—C204	177.58 (11)		

### 2-(Dinaphtho[2,1-d:1',2'-f][1,3]dithiepin-4-yl)-3,3-dimethylbutan-2-ol (2) Hydrogen-bond geometry (Å, º)

*Cg*2 and *Cg*4 are the centroids of the C105–C110 and C205–C210 rings, respectively.

**Table:**

*D*—H···*A*	*D*—H	H···*A*	*D*···*A*	*D*—H···*A*
O2—H2···S1	0.84	2.52	2.9942 (14)	117
C203—H203···*Cg*2^i^	0.95	2.60	3.4606 (19)	151
C103—H103···*Cg*4^ii^	0.95	2.93	3.443 (2)	115

Symmetry codes: (i) −*x*+1, *y*+1/2, −*z*+1/2; (ii) −*x*+3/2, *y*−1/2, *z*.
